# Freshwater mussels (Unionidae) brought into captivity exhibit up-regulation of genes involved in stress and energy metabolism

**DOI:** 10.1038/s41598-021-81856-7

**Published:** 2021-01-26

**Authors:** Ieva Roznere, Brandon T. Sinn, Marymegan Daly, G. Thomas Watters

**Affiliations:** 1 Department of Evolution, Ecology, and Organismal Biology, The Ohio State University, Columbus, OH 43210 USA; 2 Department of Biology and Earth Science, Otterbein University, Westerville, OH 43081 USA

**Keywords:** Molecular biology, Zoology

## Abstract

Approximately two thirds of freshwater mussel species in the United States and Canada are imperiled, and populations are declining rapidly. Translocation and captive management are commonly used to mitigate losses of freshwater mussel biodiversity, but these conservation tools may result in decreased growth and increased mortality. This study uses RNA-Seq to determine how translocation into captivity affects gene expression in *Amblema plicata*. Mussels were collected from the Muskingum River in Ohio, USA and brought into a captive holding facility. RNA was extracted from gill tissue 11 months post translocation from mussels in captivity and the Muskingum River on the same day. RNA was sequenced on an Illumina HiSeq 2500, and differential expression analysis was performed on de novo assembled transcripts. More than 1200 transcripts were up-regulated in captive mussels, and 246 were assigned functional annotations. Many up-regulated transcripts were involved in energy metabolism and the stress response, such as heat shock proteins and antioxidants. More than 500 transcripts were down-regulated in captive mussels, and 41 were assigned functional annotations. We observed an over-representation of down-regulated transcripts associated with immune response. Our work suggests that *A. plicata* experienced moderate levels of stress and altered energy metabolism and immune response for at least 11 months post translocation into captivity.

## Introduction

Freshwater mussels (family Unionidae) are among the most imperiled groups of animals in the world^1–3^. Over the past 200 years, these animals have suffered from habitat destruction and alteration such as river channelization and impoundment, overharvesting, pollution, invasive species, and the more recent enigmatic declines^4–7^. Approximately two thirds of the 300 species found in the United States and Canada are now classified as endangered, threatened, or vulnerable, and 10% have become extinct^6^. As numbers of freshwater mussels continue to decline, conservation efforts have increased in order to protect existing populations^6,8^. Many of these efforts involve translocating mussels to other habitats or into captivity in propagation and research facilities. In the United States, over a dozen federal and state facilities, zoos, and aquariums specialize in freshwater mussel propagation as recommended in species recovery and conservation plans^8,9^.

Captive holding of animals in zoos or other specialized facilities is a common and important conservation strategy^10,11^. Many facilities have captive breeding programs that propagate threatened and endangered species for reintroduction to extirpated areas or augmentation of existing populations^9,12–14^. Some captive breeding programs maintain species threatened with extinction in zoos and aquaria for multiple generations to ensure their survival, with the goal of future reintroduction once threats to their existence have been removed^15^. Freshwater mussels are also often translocated to different habitats or brought into captive holding facilities for temporary refuge and propagation to mitigate damage from in-stream construction activities, toxic river spills, and zebra mussel infestations^16–19^. These ex situ management efforts are powerful tools used to maintain or increase biodiversity, but may also cause stress for the animals, making them more vulnerable to factors that directly contribute to translocation failure, such as starvation, disease, and reduced reproductive capacity^20^.

While some studies have found only minimal effects of translocation on survival of freshwater mussels^21,22^, this practice often results in increased mortality and/or reduced growth rates^19,23–25^. However, models of future extinction rates predict that, without effective intervention, more than 40% of North American freshwater mussel species will become extinct over the next 100 years^26^. Translocation and captive breeding programs continue to be crucial and necessary tools in freshwater mussel conservation, and thus efforts should focus on making these techniques as successful as possible. One of the major factors limiting conservation success is our limited knowledge of freshwater mussel health and disease^27^. Because mussel physiology is understudied, the exact roles of potential causes for population declines (e.g., habitat destruction, pollution) are poorly understood^27^. The effects of captivity on mussel physiology and appropriate health assessment techniques are also unclear^28^.

In a previous study, we found that translocation of the freshwater mussel *Amblema plicata* (Threeridge), whether to another river system or into captivity, induces a similar general stress response, characterized by decreased levels of metabolites involved in amino acid, polyamine, methionine, and nucleotide metabolism^29^. Because mussels exhibited changes in primary metabolic pathways up to a year post-translocation, it is reasonable to infer that these metabolic changes were accompanied by changes in gene expression. However, there are few genomic resources available for freshwater mussels^30^, and while some studies have looked at the effects of environmental stressors on gene expression of freshwater mussels^31–33^, none have described the effects of translocation to other habitats. We sequenced and characterized the transcriptome of the freshwater mussel *Amblema plicata* to establish the necessary molecular resources to assess transcriptomic changes in response to various experimentally-induced environmental stressors in this tolerant species^34^. Here, we leverage the power of RNA-Seq in a comparative transcriptomic framework to determine how translocation into captivity changes the gene expression profile of *A. plicata* so that we can better understand the physiology of *A. plicata* and the effects of this common conservation technique. Specifically, the study objectives were to determine whether *A. plicata* experience stress after 11 months in captivity, and whether changes in gene expression reveal how biological pathways are governed.

## Methods

### Sample collection

Four adult specimens of *A. plicata* were collected from the Muskingum River in Devola, Ohio, USA, below Devola Lock and Dam #2 (39.468703 N, − 81.489303 W) on 19 September 2014. None of the mussels were gravid at time of collection. Upstream of this location is mostly valley with limited agriculture in the floodplain and a few small towns. The river is impounded by a series of low-head dams and associated locks. The drainage area covers 7440 mi^2^ and average flow during the study period was 8280 ft^3^/s^35^. This species was chosen because it is common, not listed by state or federal agencies, and found in a wide variety of habitats^36^. Mussels were transported (~ 3 h) to the Columbus Zoo and Aquarium Freshwater Mussel Conservation and Research Center (FMCRC) in Powell, Ohio, USA in insulated coolers filled with water from the collection site and equipped with an air pump. At the FMCRC, mussels were housed in a 1.5 × 1 m tank with approximately 5 cm of gravel sediment and 10 cm of water. The facility is supplied with stream-side, flow-through water from the Scioto River, which drains extensive agricultural areas from tributaries and flows through a few large towns. The drainage area covers 980 mi^2^ and average flow during the study period was 1060 ft^3^/s^35^. Because mussels were supplied with water from a natural river source, no additional food was provided. Gill tissue was sampled on 7 August 2015 (~ 11 months post-translocation) from each of 4 mussels in captivity (treatment group). That same day, gill tissue was also sampled from 3 mussels collected in the Muskingum River (control group). Less than 30 mg of gill tissue was biopsied from each individual to ensure a non-lethal sampling procedure. Each tissue sample was placed in a 2-mL RNase-free cryotube, snap frozen in liquid N_2_, and stored at − 80 °C. The Institutional Animal Care and Use Committee does not regulate the use of freshwater mussels.

### RNA extraction and sequencing

Tissue samples were mechanically disrupted and homogenized using a Mini-BeadBeater-8 (BioSpec Products, Bartlesville, Oklahoma). RNA was extracted using an RNeasy Mini Kit (Qiagen, Valencia, California). RNA concentration and integrity were measured using an Agilent 2100 Bioanalyzer (Agilent Technologies, Santa Clara, California) at The Ohio State University Comprehensive Cancer Center (Columbus, Ohio). All samples had an RNA Integrity Value (RIN) of > 7.5. RNA-Seq library preparation and sequencing were performed by the Molecular and Cellular Imaging Center at the Ohio Agricultural Research and Development Center (Wooster, Ohio). RNA-Seq libraries were prepared using the Illumina TruSeq Stranded mRNA Library Prep Kit (Illumina, San Diego, California) and sequenced on the Illumina HiSeq 2500 Sequencer (Illumina, San Diego, California) as 100 base pair (bp) paired-end reads.

### Transcriptome assembly, differential expression analysis, and annotation

Quality of sequencing data was assessed with FastQC (version 0.11.5; http://www.bioinformatics.babraham.ac.uk/projects/fastqc/). Quality and adapter trimming was performed using the BBMap package BBDuk (https://sourceforge.net/projects/bbmap/) (with options ktrim = r, k = 23, mink = 11, tpe, tbo, qtrim = rl. trimq = 15, maq = 20, minlen = 70). Only reads with an average Phred quality score of 20 and a minimum length of 70 bp were used in downstream analyses. De novo assembly of trimmed reads was performed using Trinity (version 2.6.6)^37^ using default parameters. The assembly was filtered using TransRate (version 1.0.3)^38^ and redundant transcripts (with a minimum similarity of 95%) were removed using cd-hit-est (version 4.7)^39^. To assess the quality of the final transcriptome assembly, the percentage of raw reads represented in the assembly was estimated by mapping with Bowtie2 (version 2.3.4.1)^40^ and assembly completeness according to conserved metazoan ortholog content was assessed using BUSCO (Benchmarking Universal Single-Copy Orthologs, version 3.0.1)^41^. Transcript abundance was determined using Salmon (version 0.9.1)^42^ and differential expression analysis was performed using the Bioconductor software package edgeR (version 3.16)^43^. Differentially expressed transcripts between captive and wild mussels were defined as those with a *p*-value of *p* < *0.05* and a minimum fold-change of 2.

Differentially expressed transcripts were used as BLASTx queries against the National Center for Biotechnology Information (NCBI) nonredundant (nr) database (downloaded 9 July 2018) with a word size of 6, an e-value cutoff of 1e^−5^, and a hit threshold number of 20. Functional annotation of transcripts using Gene Ontology (GO) terms and InterProScan was performed using Blast2GO (version 5.2.4)^44,45^ using default parameters. Fisher’s Exact Test was conducted to identify GO terms that are significantly over-represented in the up-regulated and down-regulated sets of genes^46^. To ensure that our discussion of differentially expressed transcripts was not affected by potential prokaryotic contamination, we filtered transcripts using Kraken2 (version 2.1.1)^47^ with a combination of its Archaea and Bacteria databases. We used a kmer length of 35 and a classification confidence threshold cutoff of 0.05.

## Results

Illumina sequencing produced 169,278,906 raw reads. The final transcriptome assembly consisted of 312,705 transcripts with a mean length of 675 bp, N50 of 1033 bp (50% of transcripts are equal to or larger than this value), and guanine-cytosine (GC) content of 35.73% (Table 1). Bowtie 2 calculated a 92.37% read alignment to the transcriptome assembly. BUSCO analysis indicated that the assembly produced 851 (87.0%) complete, 80 (8.2%) fragmented, and 47 (4.8%) missing BUSCOs. Raw data and transcriptome assemblies are archived in GenBank under BioProject PRJNA436349.

**Table 1:** Summary statistics for sequencing and transcriptome assembly.

Statistic	Value
Raw reads produced by Illumina sequencing	169,278,906
Estimate of reads used in final assembly	92.37%
Total assembled transcripts	312,705
Total assembled bases	211,028,469
Mean transcript length	675 bp
Median transcript length	383 bp
N50	1033 bp
Minimum transcript length	201 bp
Maximum transcript length	21,023 bp
GC content	35.73%

**Figure 1:**
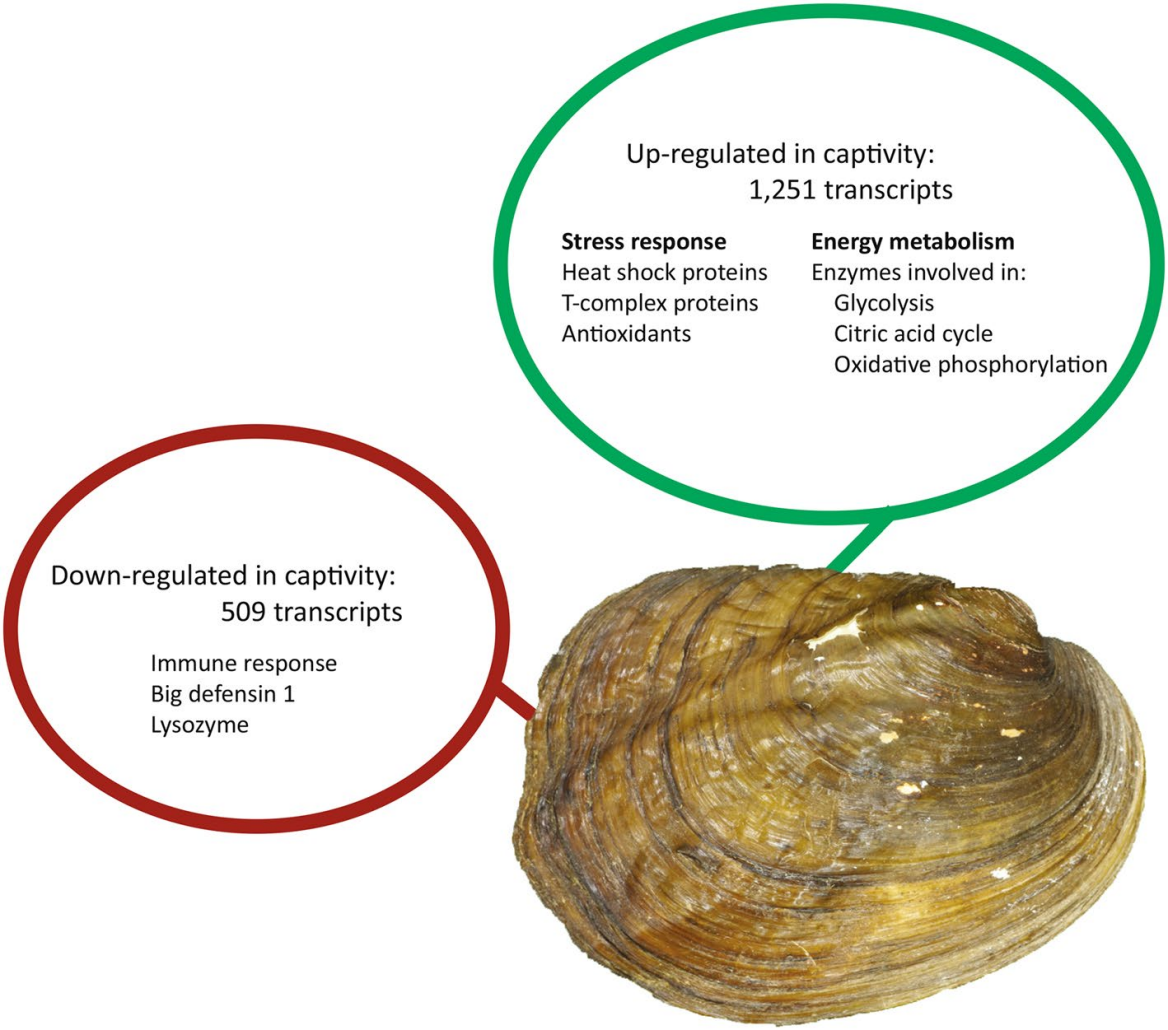
Overview of differential gene expression in *Amblema plicata* held in captivity for 11 months.

**Figure 2:**
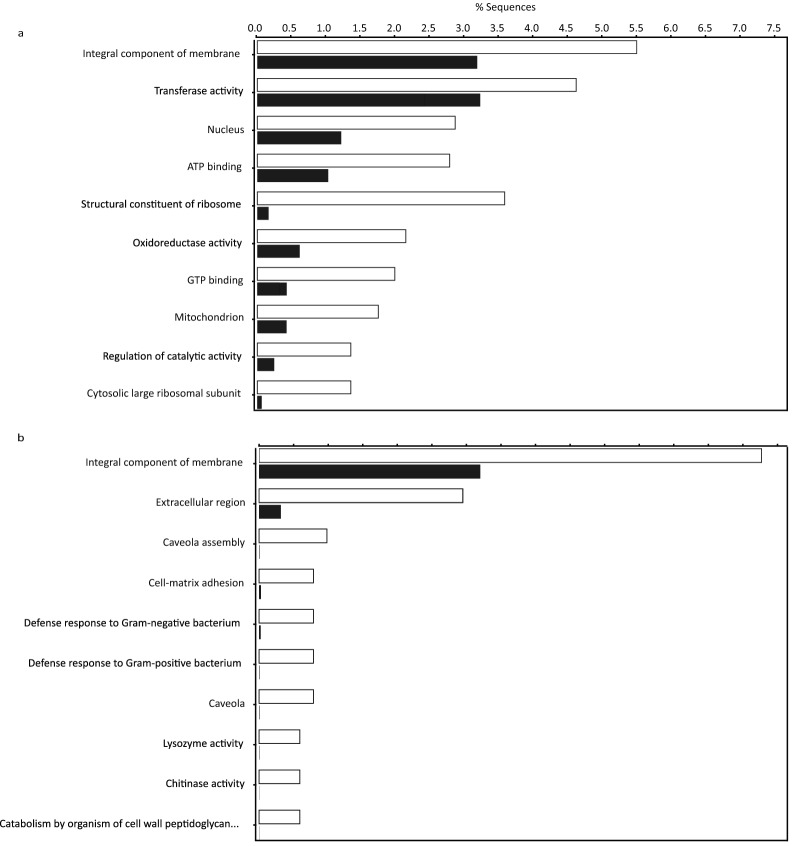
Over-represented Gene Ontology (GO) terms among (**a**) up-regulated and (**b**) down-regulated transcripts in mussels brought into captivity. White bars correspond to the transcriptome. Black bars correspond to the differentially expressed subset of transcripts.

We detected differential expression between the study populations in 1760 transcripts (Fig. 1). Greater than 70% of the differentially expressed transcripts in captive mussels were up-regulated rather than down-regulated. Of the 1251 transcripts up-regulated in translocated, captive mussels, 527 received BLAST hits and 246 were annotated. Among these, we observed a significant over-representation of GO terms associated with energy metabolism (Fig. 2a). Of the 509 transcripts down-regulated in translocated, captive mussels, 161 received BLAST hits and 41 were annotated, and there was a significant over-representation of GO terms associated with immune response (Fig. 2b). All differentially expressed transcripts and their corresponding *p*-values, false discovery rates, log fold changes, and functional annotations are provided in Supplementary Table S1. Kraken2 classified 0.17% of transcripts from the entire transcriptome as prokaryotic. Four of these transcripts were up-regulated in captive mussels, and were not included in our discussion.

### Stress response

Mussels brought into captivity showed increased expression of transcripts coding for proteins involved in the stress response (Table 2). These included many molecular chaperones such as heat shock proteins (HSPs) in the HSP90, HSP70, and HSP10 families. Many of these transcripts were assigned the GO term “ATP binding”, which was significantly over-represented among up-regulated transcripts (Fig. 2a). Other GO terms included “integral component of membrane”, “nucleus”, and “mitochondrion” (Fig. 2a). Transcripts coding for antioxidant enzymes were also found to be up-regulated and included superoxide dismutase, catalase, peroxiredoxin, and glutathione *S*-transferase (although a transcript variant of the latter was also found to be down-regulated). Over-represented GO terms that included some of these transcripts included “oxidoreductase activity” and “transferase activity” (Fig. 2a).

**Table 2:** Key transcripts differentially expressed in *Amblema plicata* brought into captivity.

Putative function	Log_2_ fold change	E-value	Gene Ontology (GO) terms
**Molecular chaperones**			
Heat shock protein 90, putative	7.44	0E0	ATP binding
Heat shock 70 kDa protein	8.30	0E0	ATP binding
Heat shock 70 kDa protein 4 isoform X1	8.91	2.71E−39	Integral component of membrane, ATP binding, nucleus
Hypoxia up-regulated protein 1	6.84	4.73E−29	Integral component of membrane, ATP binding
Heat shock protein beta-1-like	4.26	1.23E−26	
T-complex protein 1 subunit alpha	8.04	3.43E−120	
T-complex protein 1 subunit beta	7.85	4.26E−97	ATP binding
T-complex protein 1 subunit epsilon	6.58	0E0	
T-complex protein 1 subunit zeta	6.79	2.75E−170	ATP binding
T-complex protein 1 subunit theta-like	5.07	4.02E−79	
Chaperonin CPN60-2, mitochondrial	7.23	0E0	Mitochondrion
10 kDa heat shock protein, mitochondrial	6.28	4.89E−21	Mitochondrion
**Antioxidants**			
Catalase	5.30	0E0	Oxidoreductase activity
Glutathione S-transferase-like	5.56	8.42E−33	Transferase activity
Peroxiredoxin-1	6.49	1.66E−53	Oxidoreductase activity
Superoxide dismutase	7.50	1.37E−51	
**Energy metabolism**			
Triosephosphate isomerase	5.25	2.01E−60	
Glyceraldehyde 3-phosphate dehydrogenase	5.91	7.56E−131	
Enolase	7.98	3.92E−162	Nucleus
Citrate synthase	7.65	1.23E−136	Transferase activity, mitochondrion
Isocitrate dehydrogenase	7.78	3.28E−35	Oxidoreductase activity, mitochondrion
Succinate dehydrogenase [ubiquinone] iron-sulfur Subunit, mitochondrial	7.47	4.61E−114	Oxidoreductase activity, mitochondrion
Malate dehydrogenase	8.72	3.81E−113	Oxidoreductase activity
NADH dehydrogenase [ubiquinone] flavoprotein 1, mitochondrial	7.59	0E0	Oxidoreductase activity, mitochondrion
Cytochrome c oxidase subunit 7C, mitochondrial-like	6.67	2.63E−18	Oxidoreductase activity, mitochondrion
Alternative oxidase	8.90	2.50E−144	Integral component of membrane, oxidoreductase activity
**Immune response**			
Big defensin 1	− 3.56	3.24E−39	Extracellular region, defense response to Gram-negative bacterium, defense response to Gram-positive bacterium
Lysozyme	− 3.60	3.86E−66	Extracellular region, defense response to Gram-negative bacterium, defense response to Gram-positive bacterium

### Energy metabolism

Mussels brought into captivity also showed differential expression of transcripts involved in energy metabolism. Several transcripts that code for enzymes participating in glycolysis, the citric acid cycle, and oxidative phosphorylation (electron transport chain) were up-regulated in mussels brought into captivity (Table 2). Enzymes involved in glycolysis included triosephosphate isomerase, glyceraldehyde 3-phosphate dehydrogenase, and enolase. Enzymes involved in the citric acid cycle included citrate synthase, isocitrate dehydrogenase (which catalyzes the rate-limiting step), succinate dehydrogenase, and malate dehydrogenase. Enzymes involved in oxidative phosphorylation included NADH dehydrogenase (i.e., Complex I), cytochrome c oxidase (i.e., Complex IV), and alternative oxidase (AOX). Many of these transcripts were assigned the GO terms “oxidoreductase activity” and “mitochondrion”, both of which were significantly over-represented among up-regulated transcripts (Fig. 2a). Other GO terms included “integral component of membrane”, “transferase activity”, and “nucleus” (Fig. 2a).

### Immune response

Transcripts that were down-regulated included those involved in cell signaling and the immune response (Fig. 2b). The latter included the hydrolytic enzyme lysozyme and the antimicrobial peptide big defensin 1 (Table 2), both of which are members of the bivalve innate immune system^48,49^. Both of these transcripts were assigned the GO terms “extracellular region”, “defense response to Gram-negative bacterium”, and “defense response to Gram-positive bacterium”, and these were significantly over-represented among down-regulated transcripts (Fig. 2b). However, not all immune related transcripts were down-regulated. Some, such as interferon-induced protein 44, had transcript variants that were both up- and down-regulated. Others, such as toll-like receptors and peptidoglycan-recognition protein, had numerous transcripts present in the transcriptome but showed no differential expression between captive and wild mussels. Although captivity seems to have affected the immune response of mussels, the varied differential expression patterns made the exact nature of the effect unclear.

## Discussion

### Stress response

Our primary goal was to determine whether mussels brought into captivity were experiencing stress 11 months post translocation. The translocation of an organism to a new habitat is likely to change the physiological makeup of that organism for at least some period of time. Translocated organisms may need to re-establish homeostasis after being confronted with a change in their environment. Although the mussels translocated into captivity were undisturbed for 11 months, molecular manifestations of chronic stress were evidenced by the large number of up-regulated transcripts coding for various HSPs and antioxidants.

Numerous transcripts coding for heat shock proteins (HSPs) were up-regulated in captive mussels (Table 2). HSPs were first discovered to be induced in response to heat shock^50,51^, but were subsequently determined to play a wider cytoprotective role against various stressors^52^. For example, in mollusks, HSP expression is induced in response to xenobiotic contaminants^53^, hypoxia^54^, elevated CO_2_^55^, and pathogen infection^56^. The up-regulation of numerous transcripts coding for HSPs in captive mussels studied here indicates that these animals were experiencing stress 11 months post translocation.

The up-regulation of transcripts coding for antioxidant enzymes suggests that captive mussels were also subjected to elevated levels of reactive oxygen species (ROS). ROS are natural byproducts of aerobic metabolism, however stress can disrupt the balance between production and elimination and lead to increased ROS levels, i.e. oxidative stress, and consequently damage to lipids, proteins, and DNA^57^. Because ROS are eliminated by antioxidants, the up-regulation of numerous antioxidant transcripts indicates that captive mussels were experiencing oxidative stress. The increased presence of antioxidants could be caused by a wide variety of stressors, similar to our findings regarding heat shock proteins. Environmental factors that may cause oxidative stress in aquatic organisms include changes in temperature, oxygen availability, metal ions, and pollutants^58^. For example, Gillis et al.^59^ found that freshwater mussels living downstream of an urban area were exposed to complex mixtures of contaminants and exhibited higher levels of oxidative stress. The FMCRC is supplied with flow-through water from the Scioto River, mimicking ambient temperature and food availability. The Scioto and Muskingum Rivers are both large river systems but episodic or persistent variation in any of the aforementioned environmental variables could potentially cause stress in translocated mussels.

### Energy metabolism

Mussels brought into captivity exhibited up-regulation of numerous transcripts involved in the major energy metabolism pathways: glycolysis, citric acid cycle, and oxidative phosphorylation. The increased expression of these enzymes, some of which regulate rate-limiting steps, suggests that captive mussels experienced increased energy demand. One of the up-regulated transcripts, AOX, is a terminal oxidase in the electron transport chain that provides an alternative route for electrons typically passing through Complex III and IV^60^. The simultaneous up-regulation of cytochrome c oxidase (Complex IV) suggests that both the typical and alternative electron transport routes are engaged to maximize ATP production. Although the AOX route is less efficient, it can reduce the production of reactive oxygen species^61^ and, therefore, the up-regulation of this enzyme may be a means to limit oxidative stress during increased metabolic activity.

Energy balance plays an important role in animal survival and stress tolerance. Organisms allocate energy resources between various biological processes such as maintenance, growth, activity, and reproduction^62^. Sokolova et al.^63^ propose that under moderate levels of environmental stress, metabolism may increase to meet additional energy needs, while severe levels of stress tend to cause metabolic depression. In the prior scenario, long-term survival of the organism is possible, while in the latter scenario it is not. The up-regulation of energy-producing pathways in *A. plicata* brought into captivity suggests that these mussels are experiencing chronic levels of moderate stress. Increased ATP production may be necessary for the synthesis of the aforementioned stress proteins, antioxidants, and other compounds involved in homeostatic maintenance.

In a previous study, we relocated *A. plicata* into captivity and used metabolomics to describe changes in biochemicals throughout the following year^29^. We found that metabolites involved in energy metabolism (e.g., fructose, galactose, glucose, lactate, arabinose) and most lipids did not differ between captive and wild mussels. These results led us to conclude that energy metabolism was not affected by captivity. However, our present results show that energy metabolism is impacted by the stress of captivity. It may be that while the *levels* of metabolites in the organism remain constant, the *flux* of these metabolites through the energy pathways has increased, which may explain the up-regulation of metabolite-interconverting enzymes. However, these studies were conducted in different years and energy metabolism may have been influenced by different factors, such as temperature, natural food availability, or reproductive state.

### Immune response

Several transcripts likely coding for immune response proteins were found to be differentially expressed in mussels in captivity. Two down-regulated transcripts with important roles in the bivalve immune system were big defensin 1 and lysozyme. Defensins are antimicrobial peptides that are active against Gram-positive and Gram-negative bacteria, fungi, and viruses^64^. Defensins are the most common group of antimicrobial peptides in bivalves^49^ and big defensin 1 has been shown to be strongly induced in response to a *Vibrio* infection in the oyster *Crassostrea gigas*^65^. In addition, we found down-regulation of transcripts coding for the hydrolytic enzyme lysozyme. Lysozyme contributes to pathogen neutralization and plays an important role in bivalve antimicrobial defense^48^. In bivalves subjected to infection, lysozyme has been shown to primarily increase expression in mucosal tissues such as the mantle, gills, and digestive gland^49,66^. However, we found that not all transcripts with potential immune response functions were down-regulated in captive mussels. For example, some transcript variants coding for interferon-induced protein 44, a protein involved in bivalve anti-viral response^67^, were down-regulated, while others were up-regulated. Toll-like receptors, which are widely regarded as important pattern recognition receptors involved in immune response^68^ were identified in our transcriptome but did not exhibit differential expression. Mussels in captivity might experience less infection or immune stress than those in the wild, and so have lower levels of expression for transcripts that respond to infection. This could also reflect differences between the intensity of immune-provoking agents (bacteria, parasites, chemicals) between the Muskingum and Scioto Rivers, or simply reflect the “snapshot in time” nature of transcriptomes, if the reference transcriptome from a mussel in the wild happened to be fighting infection and thus experiencing relatively high expression of immune response proteins. However, it may also be that chronic stress causes captive mussels to have lower expression of immune proteins because stress or nutritional levels demand that energy be allocated elsewhere. Although up-regulation versus down-regulation varied by transcript, captivity clearly had an effect on the mussel immune system.

The effect of translocation stress on the freshwater mussel immune system is worthy of further investigation. In addition to differential expression of immune related transcripts, we noticed that the subset of up-regulated transcripts had a surprising number of BLAST hits to ciliates such as *Paramecium* and *Tetrahymena*. It may be that the compromised immune system of the chronically stressed mussels allowed for increased bacterial growth on the mucosal gill surface and thus to greater concentration of bactivorous ciliates. Water in the facility may also carry a higher ciliate load compared to that of the natural river environment from which these mussels were collected. Most studies have described no adverse effects of ciliate presence in freshwater mussels^69,70^. However, it is important to note that genomes of freshwater mussels are poorly understood, and these BLAST hits could be due to a paucity of comparative genomic resources and knowledge of horizontal gene transfer in bivalves^71^. Only 19% of our transcriptome received BLAST hits, leaving most assembled transcripts with no known putative function, a direct consequence of a lack of fully annotated freshwater mussel genomes. Nevertheless, future work on the interplay between freshwater mussel immune response and microbiome could provide useful information about stress-induced changes in freshwater mussel health.

## Conclusion

The freshwater mussel *A. plicata* is a common species found in a wide variety of habitats, and it is likely that rare species with narrow habitat requirements might experience more severe levels of stress after translocation. Yet conservation projects involving translocation usually focus on rare and endangered species. *Amblema plicata* experienced stress in captivity 11 months post translocation, as evidenced by increased expression of transcripts in gill tissue coding for heat shock proteins, antioxidants, and immune response. Although we only collected a small amount of gill tissue in order to maintain a non-lethal sampling procedure, more insight could be gained in future studies by sampling the whole soft body or various other tissue types, since different tissues may show variable responses^55^. For example, analysis of mantle tissue might provide more information about changes in expression of genes regulating growth and mantle formation, and analysis of foot and adductor muscles could provide more insight into energy use and demand by other organs. Analysis of gene expression in other tissues could also provide more information about the distribution of the stress response among various organs. However, sampling multiple tissue types or internal organs could be lethal to the organism, an important consideration especially when working with threatened or endangered species. In our study, increased expression of transcripts involved in energy metabolism indicated that mussels were experiencing chronic, moderate stress. Although we estimate that the studied mussels were experiencing only moderate stress, we find our ability to detect stress 11 months after translocation to be concerning, especially because *A. plicata* is usually considered a tolerant species. Furthermore, a chronically stressed mussel may not grow at a normal rate or reproduce successfully, both of which are important factors in successful propagation projects. In this study, we did not attempt to isolate the effects of a specific stressor (e.g., food supply, substrate composition, water temperature) but recognize that mussels moved into captivity are presumably exposed to changes in multiple environmental variables, and multiple stressors often have synergistic and unpredictable effects on gene expression^72^. Differences in water composition between the Muskingum and Scioto rivers could also contribute to stress observed in our translocated animals, and future work should consider the ways that differences between these systems might contribute to the stress experienced by translocated mussels. Because wild *A. plicata* live in both the Muskingum and Scioto Rivers, neither system is inherently unsuitable for this species; the stress response we see in the captive mussels is thus probably related to translocation or captivity.

Our results highlight the explanatory power of comparative transcriptomics and provide a powerful foundation upon which to study the effects of stress on mussel physiology. For example, our results can be used in future work to explore more granular changes in metabolic pathways we found to be affected by stress of captivity. Additionally, we can use transcriptomic tools to study how changing specific variables in captivity affects the organism’s stress response. In this way, we can not only learn more about mussel physiology but also improve conditions in research and propagation facilities. Captivity is an important aspect of freshwater mussel conservation and improving our understanding of the effects of stressors on mussel health is crucial if we are to save these endangered animals. Beyond captivity induced changes, transcriptomic tools can be used to study responses to a wide variety of environmental stressors to advance our understanding of freshwater mussel physiology.

## Supplementary Information

Supplementary Information
